# Machine learning-based text mining for cutaneous myiasis and potential value of an accidental maggot therapy for complicated skin and soft tissue infection with sepsis

**DOI:** 10.3389/fcimb.2025.1568563

**Published:** 2025-05-06

**Authors:** Zhiyuan Zhou, Chaoran Yu, Danhua Yao, Zhen Wang, Yuhua Huang, Pengfei Wang, Weimin Wang, Yousheng Li

**Affiliations:** Department of General Surgery, Shanghai Ninth People’s Hospital, Shanghai Jiao Tong University School of Medicine, Shanghai, China

**Keywords:** bibliometric, diabetes, cutaneous myiasis, latent Dirichlet allocation, soft tissue infection

## Abstract

**Background:**

Cutaneous myiasis, one of the most frequently diagnosed myiasis types, is defined as skin or soft tissue on a living host infested by dipterous larvae (maggots). However, bibliometric analysis of this disease remains sparse. Machine learning techniques and updated publications provide an opportunity for such an investigation.

**Materials and methods:**

All the studies were retrieved from PubMed and were processed using R software in the bibliometric analysis and latent Dirichlet allocation (LDA) topic modeling. Furthermore, the clinical management of two diabetes patients with serious soft tissue infection-associated sepsis was analyzed.

**Results:**

A total of 211 results were retrieved and 50 topics relevant to cutaneous myiasis were determined by the LDA algorithm. The topics of uncommon fly species, nasal infestation, and physician discussion of cutaneous myiasis were consistently common over the last 20 years. Case report remains one of the key features in myiasis. Four major clusters were identified, i.e., case report related, disease type and development, travel in the tropics, and skin disease. To further delve into clinical practice, the clinical features of two patients with soft tissue infection-related sepsis were demonstrated, and a distinct beneficial role of myiasis was found. The levels of white blood cell, blood glucose, and C-reactive protein in the case with cutaneous myiasis were more stable than the other case without cutaneous myiasis but with sepsis shock.

**Conclusion:**

Maggot debridement therapy may be a promising treatment and beneficial for soft tissue infection-related sepsis. The model analysis of maggot therapy and its clinical advantages shows increasing research value and possible application in future clinical practice.

## Introduction

Cutaneous myiasis, one of the most frequently diagnosed myiasis types, is defined as skin or soft tissue of live vertebrate hosts infested by dipterous larvae (maggots), with three major clinical types: furuncular, migratory, and traumatic myiasis (Mandell et al., 1979; Caumes et al., 1995; McGraw and Turiansky, 2008; Robbins and Khachemoune, 2010). The infesting fly larvae feed on the dead or living tissue of the host for a certain period. The disease is commonly found in both veterinary and travel medicine, particularly in tropical climates. Indeed, it has been recorded as one of the most common skin diseases associated with travel (Caumes et al., 1995). Although myiasis is a global disease, an increasing number of reported cases were found to be associated with travel and developed countries (Solomon et al., 2016).

As the number of investigations increases, it is essential to delineate the developmental trajectory of accumulating research studies in cutaneous myiasis, aiming for a research landscape to screen hotspots or emerging fields or to navigate future studies in association with multidisciplinary treatment. Of note, most publications associated with cutaneous myiasis either relate to case reports or systemic reviews due to the investigatory nature of this disease (Schüpbach et al., 2022; Albano et al., 2023; Alsaedi et al., 2023; Mohsin et al., 2023; Azarmi et al., 2024).

Based on publications retrieved from the last 20 years, we performed a machine learning-based text mining and bibliometric analysis for cutaneous myiasis. The clinical significance of myiasis therapy, also called maggot therapy, was medically valued at least five hundred years ago but this value has risen again during the last decade due to its profound efficacy, simplicity, and safety in the treatment of dead tissues when the performance of antibiotics fails to medical expectation. Nonetheless, the specific therapeutic function and target diseases are yet to be detailed. Thus, in this study, we also present a clinical report of two cases with severe soft tissue infections to elucidate the association between cutaneous myiasis and alleviated infection.

In this study, key research topics and interactions were found using machine learning techniques and bibliometric analysis, and the potential clinical value of maggot therapy was identified for soft tissue infection-induced sepsis, providing insightful contributions to both clinical practice and research fields.

## Materials and methods

All the publications concerning cutaneous myiasis were retrieved from the PubMed.gov website (https://www.ncbi.nlm.nih.gov/pubmed/) with the following search terms: myiasis (Title/Abstract) AND cutaneous (Title/Abstract) AND English (Language) AND 2001-2021 (Year Published). Search results included the title, abstract, MeSH (Medical Subject Headings) terms, and others (**Supplementary Figure S1**). All included publications were processed by bibliometric analysis and latent Dirichlet allocation (LDA) for topic modeling. Topic modeling is defined as an unsupervised classification of words retrieved from publications. This method provides a reliable way to automatically process and organize large collections of electronic publications, helping to discover rising themes or hotspots or categorize the collection. LDA, as one of the most commonly used methods in topic modeling, is a probabilistic generative model for text corpora, first introduced by David Blei and his colleagues in 2003.

R software (version 4.1.1) was employed for data cleaning and processing and the visualization of results (https://www.r-project.org/) (Ihaka and Gentleman, 1996; Hoffman et al., 2010; Aria and Cuccurullo, 2017; Jelodar et al., 2019; Liu et al., 2023).

In addition, two diabetes patients with serious cutaneous wounds were admitted to the Emergency Department of the Shanghai Ninth People’s Hospital, Shanghai Jiao Tong University School of Medicine between April and May 2022. Both patients received standard clinical therapy, and clinical data were retrieved with the patients’ consent.

## Results

### Bibliometric findings

**Figure 1:**
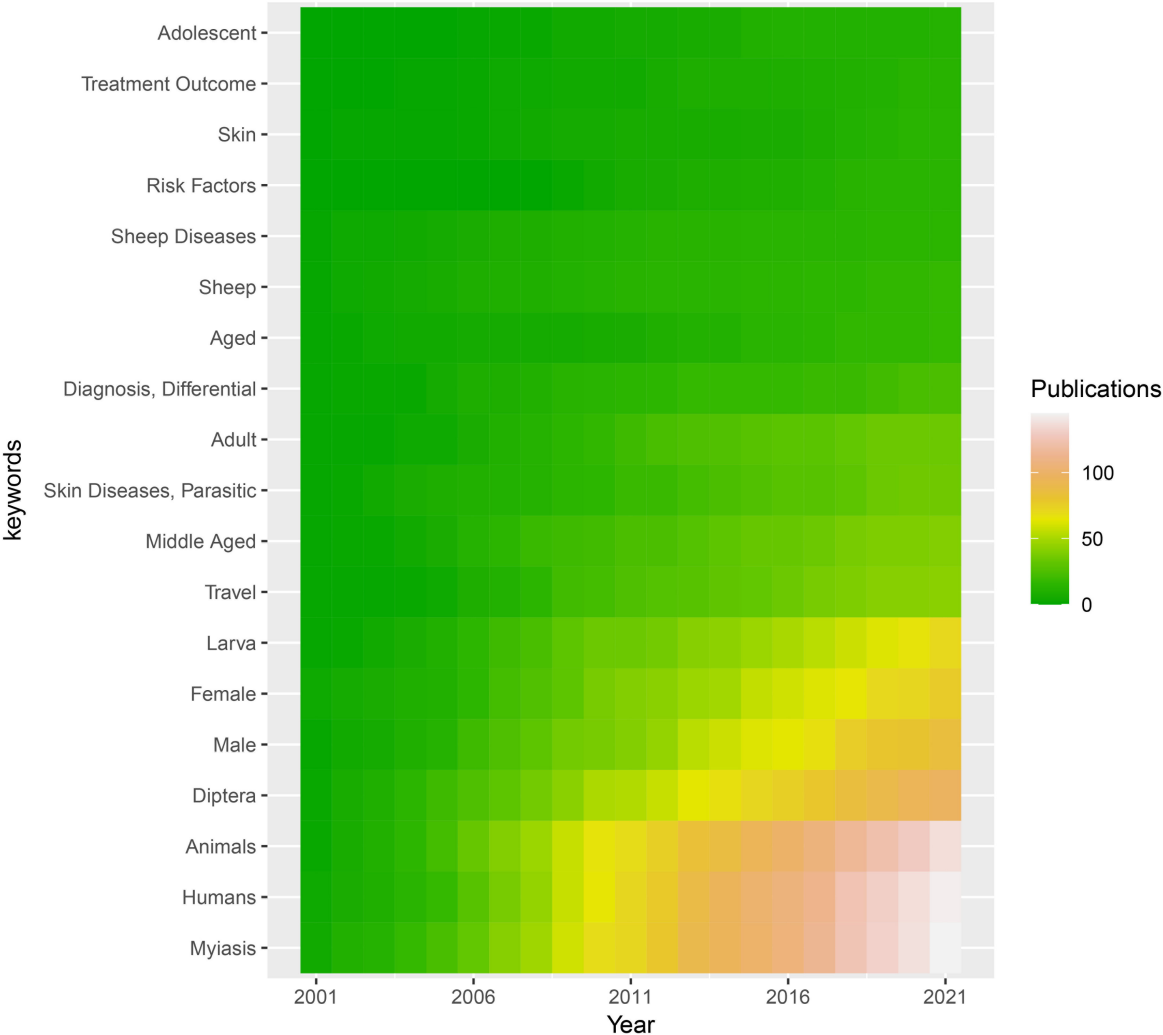
MeSH (Medical Subject Headings) relating to myiasis studies retrieved from the Pubmed (https://www.ncbi.nlm.nih.gov/pubmed/). The top 20 keywords are displayed from 2001 to 2021. Dark blue indicates a low number of publications and light blue indicates a high number of publications. .

Using the search terms, a total of 211 results were identified from PubMed. After analysis of the MeSH terms, the top twenty were shown in order. The keywords myiasis, humans, animals, Diptera, male, female, larva, and travel noticeably increased from 2001 to 2021 (**Figure 1**).

### Topic modeling results

**Figure 2:**
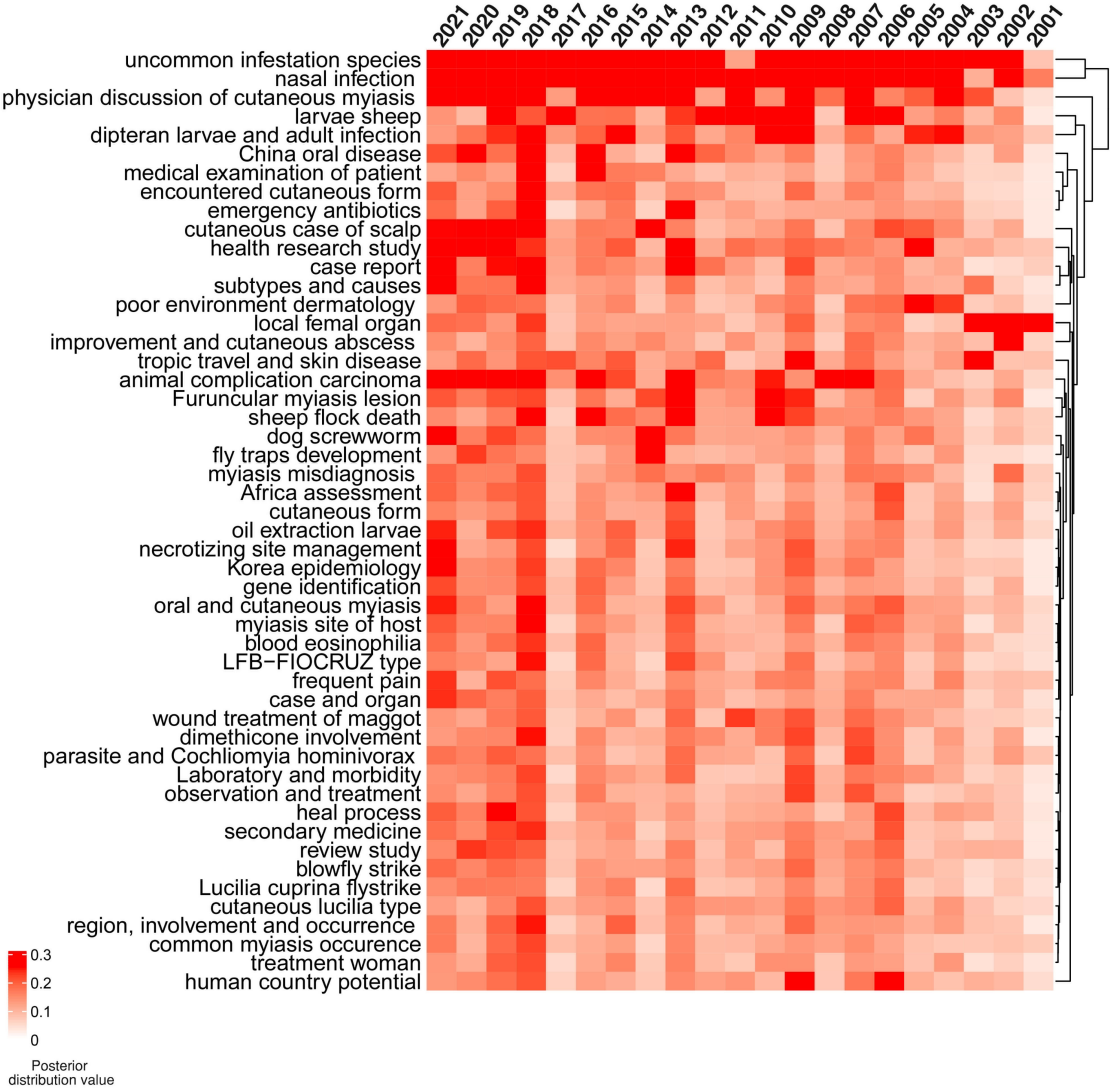
Distribution values of latent Dirichlet allocation (LDA) for 50 topics from 2001 to 2021. The topics of uncommon infestation species, nasal infection, and physician discussion of cutaneous myiasis were consistently intensively studied during the last 20 years. The topics of case report, subtypes, and causes were found in 2021. Red indicates a high value and white indicates a low value.

A total of 50 topics relevant to cutaneous myiasis were determined by the LDA algorithm from the publications’ abstracts. Each specific topic enabled a more detailed perspective on the growth in this field. The topics of uncommon fly species, nasal infection, and physician discussion of cutaneous myiasis were consistently common over the last 20 years (**Figure 2**). All the topics were visualized by heatmap and clustered based on a posterior distribution value. Therefore, uncommon infestation species, nasal infection, and physician discussion of cutaneous myiasis showed close association over the years, and all had a high value across the last 20 years. Furthermore, 2006–2007, 2009, 2013, 2016, and 2018–2021 showed higher topic distribution values than the rest.

**Figure 3:**
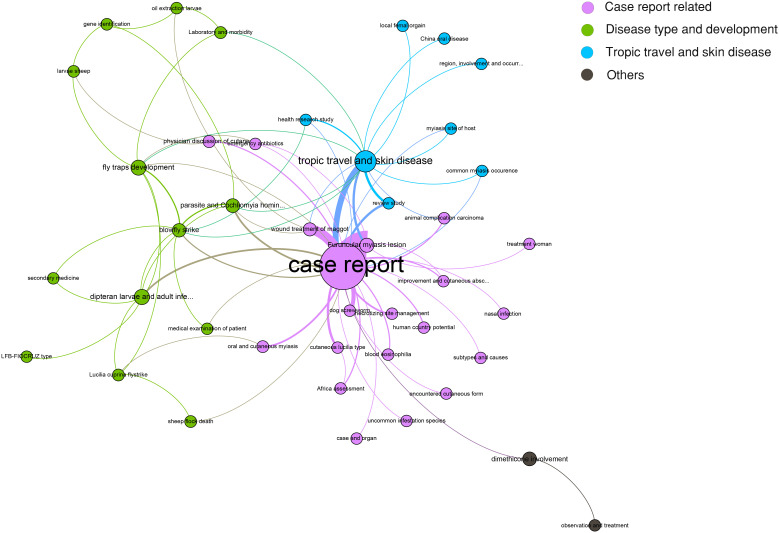
Latent Dirichlet allocation (LDA) research topic cluster network. Four clusters were determined, including case report-related (pink), disease type and development (green), tropic travel and skin disease (blue), and others (black).

However, a specific association among the topics was not clarified. Therefore, a network for topic similarity and clustering analysis was established. Remarkably, the 50 topics were categorized into four major clusters. However, three out of the four were remarkable, including case report-related, disease type and development, tropic travel, and skin disease. The central role of the case report was highlighted (**Figure 3**).

Admittedly, given the rarity of the disease, most of the publications relating to cutaneous myiasis were either related to a case report or a systemic review. Consistently, our LDA model also highlighted the central role of the case report. Therefore, we retrospectively analyzed two cases with soft tissue infection, one with myiasis and the other without, to compare the infection features between them.

### Clinical case comparisons

**Figure 4:**
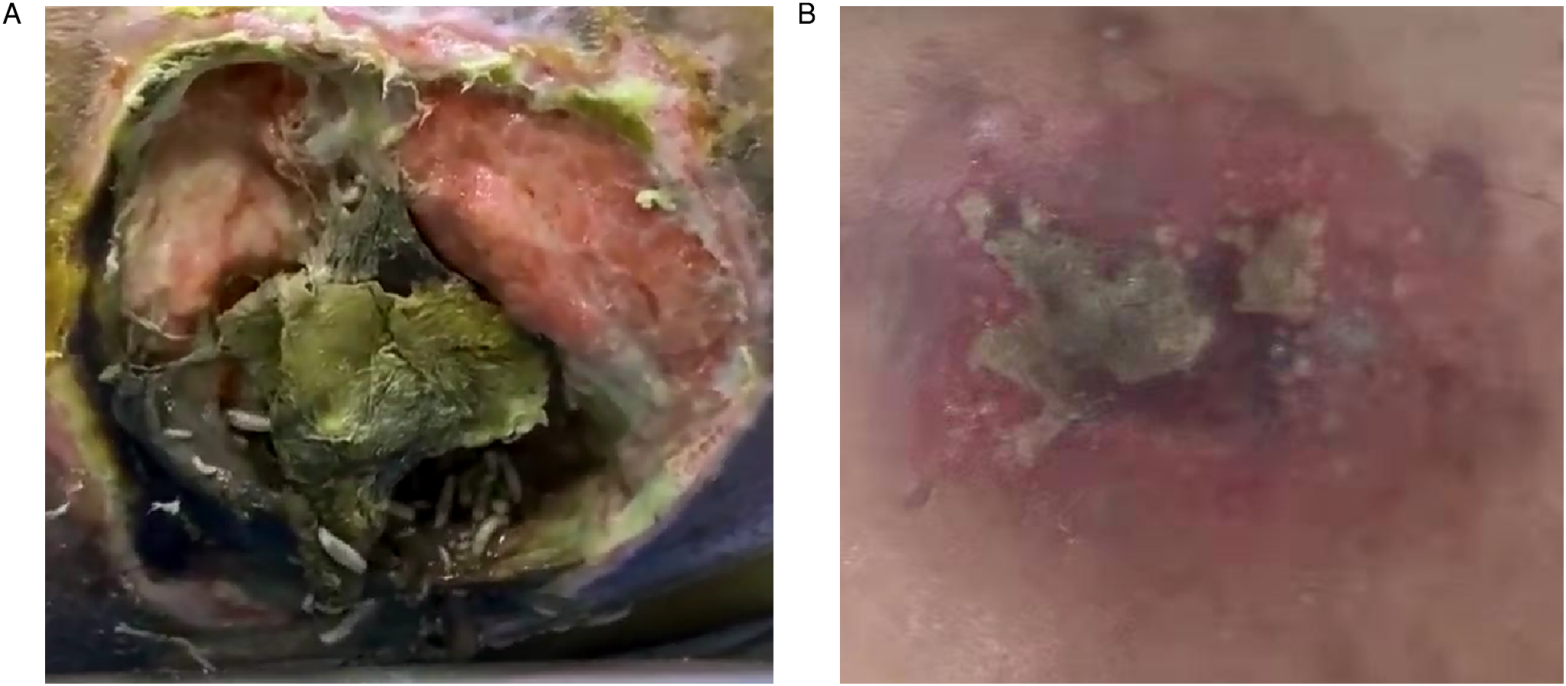
Two patients with cutaneous wounds and long-term diabetes were presented in this article. **(A)** A 70 year-old woman presented with a massive decubitus ulcer infested with maggots and necrotizing soft tissues at the lumbosacral area; **(B)** a 51 year-old man presented with a massive wound infection in neck and back area.

A 70-year-old paraplegic woman with decades of diabetes and years of being bedridden was admitted to the emergency department with a suspected case of fever, sepsis, and a massive decubitus ulcer. On examination, the woman was found to have been suffering from a massive decubitus ulcer for 2 months, resulting from long-term pressure sores, with a sleepy, sweaty, and afebrile condition. When the covering tissue was debrided, the decubitus ulcers showed hundreds of larvae crawling within, with a measured ulcer area of 10cm×10cm and necrotizing soft tissue approximately 3–4 times larger, across the lumbosacral area (**Figure 4A**). Initial visual evidence indicated that it may belong to *Calliphora Calliphoroides*, *Calliphoridae*. Similarly, a 51-year-old man with long-term diabetes who had been suffering from a massive cutaneous wound for 1.5 months was admitted but without myiasis. He was diagnosed with a confirmed case of massive neck/back wound infection with severe septic shock and unstable vital signs. On examination, the ulcer neck/back wound was approximately 5cm×10cm with necrotizing tissue and no sign of fly larvae (**Figure 4B**).

**Figure 5:**
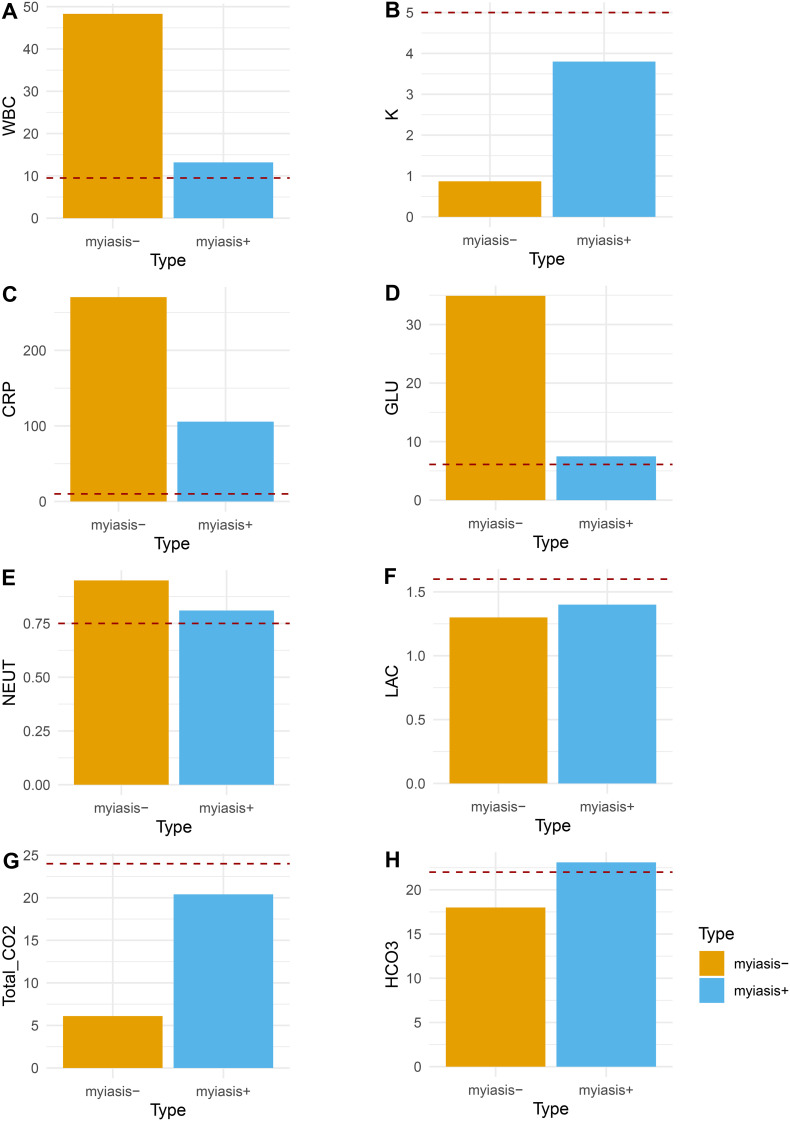
Comparison of clinical variables between the patient with maggots and the patient without maggots. **(A)** White blood cell count (WBC, 10×9/L); **(B)** blood potassium (K, mmol/L); **(C)** C-reactive protein (CRP, mg/L); **(D)** blood glucose (GLU, mmol/L); **(E)** blood neutrophils percentage (NEUT, %); **(F)** blood lactate (LAC, mmol/L); **(G)** CO2 blood test (Total_CO2, mmol/L); **(H)** Bicarbonate (HCO3, mmol/L); red dash line indicated normal value threshold.

Laboratory tests and initial vital signs indicated that the case with cutaneous myiasis may have suffered from sepsis, with comparable low blood pressure, elevated heart rate, and abnormal levels of white blood cells (WBCs; 13.19×10^9/L), blood glucose (GLU; 6.8 mmol/L). and C-reactive protein (CRP; 105.65 mg/L). However, the case without cutaneous myiasis presented with sepsis and shock, with unstable vital signs, low potassium (K+, 0.87 mmol/L), extremely high levels of glucose (34.9 mmol/L), CRP of 270.43 mg/L, and WBC of 48.30×10^9/L (**Figure 5**). Furthermore, the total CO_2_ and HCO_3_ were found to be comparably high in both cases. Taken together, the case with myiasis might have exhibited a lower level of sepsis.

**Figure 6:**
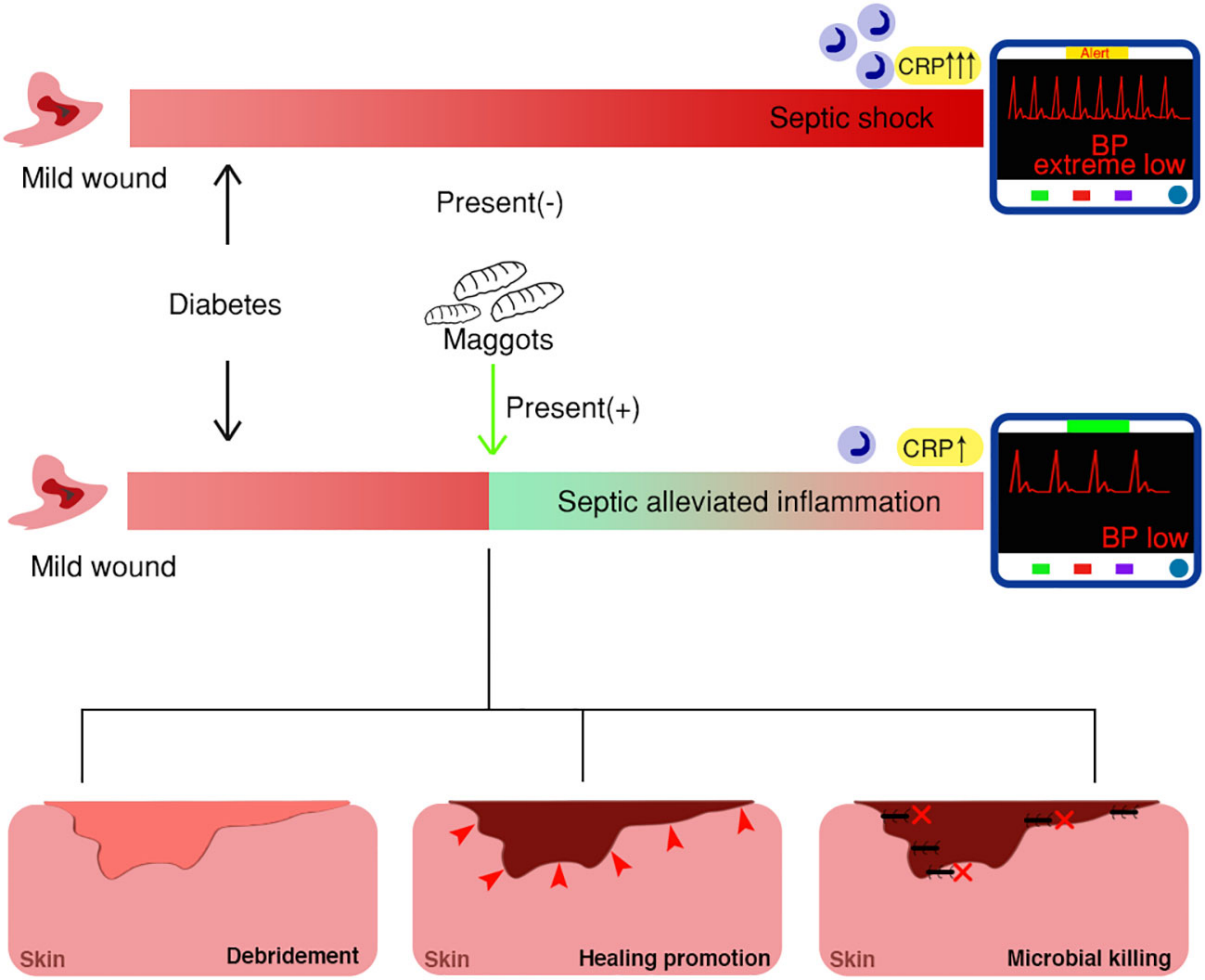
Schematic model of maggot therapy for diabetic wounds with mechanisms, depicting distinct clinical outcomes with or without maggot presentation.

Of note, based on the reported case with accidental maggot therapy and the other case without, we propose a hypothesis that medicinal maggots may have beneficial effects on sepsis associated with wounds (**Figure 6**).

## Discussion

In this study, a machine learning-based algorithm was employed for text analysis of publications retrieved from the past 20 years. A total of 50 topics were revealed but common topics were uncommon fly species, nasal infestation, and physician discussion of cutaneous myiasis. However, the analysis indicated that “case report” was the key term among all the topics. The reason could be that the case-specific and comparatively rare incidence of cutaneous myiasis compared to other malignancies makes it more feasible for case reports to be the most effective approach for studies of cutaneous myiasis in humans.

Cutaneous myiasis can be classified into three types: furuncular, creeping, and traumatic myiasis. Furuncular myiasis is commonly diagnosed in tropical America, such as Mexico and Central America (Goddard, 1996). The treatment of furuncular myiasis includes numerous local therapeutic remedies, such as pork fat, mineral oils, glue, and petroleum jelly, resulting in the suffocation of the maggots (Brewer et al., 1993; Boggild et al., 2002). However, some researchers were concerned that incomplete removal of infestations may result in granuloma formation as a portion of the larvae may require surgical extraction (DeFilippis and Leite, 1997). For creeping myiasis, as humans are not the usual hosts, the development of the larvae is limited. Therefore, they only make tunnels in the epidermis tissue. At the superficial location, the larvae can be easily removed (Manson, 1996). For traumatic myiasis, tissue debridement and irrigation are required. Removal of the larvae also requires proper anesthesia. Some reports indicated the therapeutic value of chloroform and ivermectin (Chodosh and Clarridge, 1992; Gealh et al., 2009). In summary, the various types of cutaneous myiasis have been an emerging field for future research, as such type of infection or therapy has been beneficial to both scientific and clinical practices.

Maggot debridement therapy is known worldwide to have profound efficacy in the treatment of various types of wounds (Sherman et al., 2000; Nigam and Morgan, 2016). It has been recently embraced by the healthcare community as antibiotic resistance has unprecedentedly increased. Previously, maggot therapy was used as a last-resort attempt for certain infections such as gangrene when surgery or antibiotic drugs failed, but currently, it is utilized as an adjunct therapy instead of an alternative one, covering various types of soft tissue wounds, such as traumatic, surgical-related, diabetic, or venous stasis-related wounds (Sherman et al., 2000; Sherman, 2009). However, life-threatening wound infections were previously thought not to be treated with maggot debridement as surgical intervention is more likely to be superior (Sherman et al., 2000). Of note, in this study, we reported that a septic patient with myiasis showed a lower level of inflammation than the other patient with septic and shock but without myiasis, indicating a potential protective role of myiasis in alleviating inflammation of sepsis and shock.

In this study, we hypothesized that medicinal maggots may have beneficial effects on sepsis associated with wounds (**Figure 6**). The presence of maggots may weaken the sepsis progress, mainly through three mechanisms: 1) Wound debridement or necrotic tissue elimination; 2) accelerated wound healing; 3) antimicrobial therapy. Specifically, as the infection develops, the very existence of myiasis mainly targets the necrotizing tissue, serving to reduce possible inflammation subsequently caused by massive necrotizing soft tissue infection (NSTI), such as necrotizing fasciitis. Proteolytic enzymes, such as collagenases, trypsin, or chymotrypsin-like enzymes, are produced for tissue digestion (Honda et al., 2011; Isabela Avila-Rodríguez et al., 2020; Filippis et al., 2024). For example, N-acetyl-beta-D-glucosaminidase, alpha-d-glucosidase, and alpha-D-mannosidase are all produced by *Lucilia sericata* larvae for wound debridement and further sterilization (Filippis et al., 2024). A chymotrypsin-like serine protease released by maggots is also vital to target extracellular matrix proteins, such as fibronectin and collagen, by facilitating the breaking down of necrotic tissue (Pöppel et al., 2016). In fact, digestive enzymes for wound debridement are crucial factors in inflammation control, as they determine not only tissue but also bacterial digestion, and further serve as immune response targets. Moreover, it is estimated that approximately 25mg of necrotic tissue can be eradicated by a single maggot in 24 hours (Sherman, 2002). This quick debridement considerably reduces the negative impact of inflammation or toxic reactions triggered by necrotic tissues in living tissues.

The significant antibacterial effects of maggot therapy are mainly from both physical digestion and the production of antibacterial proteins (Yan et al., 2018). Some research found that gram-positive bacteria are more sensitive to this therapy than gram-negative bacteria. Furthermore, an antifungal effect has also been observed. Several types of molecular masses have been identified among the antibacterial proteins, including lucifensin, lucilin, lysozymes, and many others (Cerovský et al., 2010; Valachova et al., 2014a; Valachova et al., 2014b; Yan et al., 2018).

Some researchers may hold the view that the accelerated growth of a wound may only be because of wound debridement (Robinson and Norwood, 1934). However, more researchers believe that ammonium bicarbonate, allantoin, and urea are responsible for the promising healing speed in wound management (Sherman, 2014).

In addition, it remains unknown whether maggot therapy has further systemic impact on the host, such as systematic antibiotic/metabolic effects or immune regulation. Interestingly, the case without myiasis also developed uncontrolled hyperglycemia, which further exacerbated the NSTI and contributed to the septic shock.

The role of myiasis, either beneficial or harmful to humans and animals, has always been one of the rising multidiscipline interests in research practice as previously described (Sherman et al., 2000; Hall et al., 2016). Hall et al. reported that given the inadvertent introduction and global spread, the incursion of fly larvae into many tropical and subtropical regions is notable and resulted in heavy costs (Hall et al., 2016). Eradication programs of such targets might be both difficult and expensive, as a 3-year eradication program cost approximately 80 million US dollars in Libya (Food Agric Organ (FAO), 1992). Meanwhile, Old World screwworm fly (OWSF)-associated myiasis remains one of the most common types of myiasis in India, causing serious medical problems, with human cases occurring continuously (Sankari and Ramakrishnan, 2010).

Currently, non-negative matrix factorization (NMF) and LDA are the most commonly used machine learning techniques for data analysis (Geiger and Park, 2024). The techniques share similar advantages in signal processing, text mining, and bioinformatics by decomposing non-negative data into parts for further modeling. NMF is marked by its constrained optimization problem using linear algebra algorithms, while LDA is characterized more like a generative model in statistics for text words and paraphrases, in order to analyze potential wordy topics and proportions of paragraphs. Although the predecessor of LDA, probabilistic latent semantic analysis (PLSA) is more closely related to NMF given its algorithms and similar results. LDA demonstrated more advantages for natural language processing than NMF, such as short-text corpora. Rkia et al. reported that the coherence score of LDA for natural language processing was 0.59, which was better than NMF (Rkia et al., 2024).

Up to now, many challenges in maggot therapy remain far from clearly solved, with setbacks either in diagnostic or therapeutic fields (Babazadeh et al., 2023; Sherman et al., 2024). In 2023, Babazadeh et al. reported mistreatment with maggot therapy for a diabetic foot ulcer, resulting in an amputation. In this case, the researchers intensively discussed the indication and contraindications of this case, specifically, *Pseudomonas aeruginosa* infections with tendon exposure may not favor maggot therapy. Other contraindications may include dry wounds or immunosuppressive patients with pyoderma gangrenosum (Babazadeh et al., 2023). However, the key point proposed by Dr. Sherman was sufficient attention to the case and therapy so as to fully understand the benefits and risks associated with maggot infection and maggot therapy (Sherman et al., 2024). A maggot infestation, or maggots in the wounds, is not equal to maggot therapy. Placing a non-therapeutic species into a wound would not be regarded as classic Maggot debridement therapy (MDT). Classic MDT, in fact, is controlled and performed with limited types of maggots, for example, germ-free *L. sericata, L.cuprina, and L.illustris*. Therefore, the introduction of rare myiasis with accidental therapy may greatly contribute to the field.

### Reflection and summary

In this study, maggot therapy was found to possibly alleviate the inflammation level of severe soft tissue infections and sepsis. More relevant clinical trials or cohort studies are encouraged to further delineate the specific parameters of maggot therapy, types of maggots, and in which stage maggot therapy achieves the best results. Standardization of maggot therapy will then be established, as well as future therapeutic guidelines. Given the three major mechanisms of wound debridement or necrotic tissue elimination, accelerated wound healing, and antimicrobial therapy, more molecular or enzymatic mechanisms are strongly encouraged to be researched. In the future, the therapeutic spectrum of maggot therapy may expand to sepsis with more solid clinical evidence yet to come. It also bridges the biological value of maggot therapy with the basic science of infection and sepsis.

## Conclusion

Key topics have been modeled with the increasing popularity of maggot therapy. Maggot debridement therapy may be beneficial for soft tissue infection-related sepsis.

## Data Availability

The original contributions presented in the study are included in the article/**Supplementary Material**. Further inquiries can be directed to the corresponding authors.

## Supplementary material

Supplementary Figure 1 The criteria flowchart of included publications.
